# Lemierre's Syndrome: A Comeback Story

**DOI:** 10.7759/cureus.25843

**Published:** 2022-06-11

**Authors:** Sobaan Taj, Christopher P Austin, Zaka Ahmed, Nusha Fareen, Zeeshan Chaughtai, Henna Pervaiz, Saira Chaughtai

**Affiliations:** 1 Internal Medicine, Jersey Shore University Medical Center, Neptune, USA; 2 Internal Medicine, St George’s University School of Medicine, True Blue, GRD; 3 Internal Medicine, State University of New York (SUNY) Downstate Medical Center, Brooklyn, USA

**Keywords:** septic emboli, septic thrombophlebitis, fusobacterium necrophorum, infectious disease medicine, lemierre's syndrome

## Abstract

Lemierre's syndrome is a very rare and life-threatening complication of bacterial pharyngitis and tonsillitis. Often referred to as a ‘forgotten disease’, Lemierre's syndrome has seen a rise in cases over the years secondary to increased antibiotic resistance. With the potential for multiple organ failure secondary to widespread septic emboli, Lemierre's syndrome can no longer be forgotten. Prompt initiation of treatment is needed for better patient outcomes. We describe an unusual case of a young female without any significant past medical history who presented with left-sided pleuritic chest pain several days after experiencing a sore throat.

## Introduction

Lemierre’s syndrome is an extremely rare complication of bacterial pharyngitis and tonsillitis that involves an extension of the infection that results in septic thrombophlebitis of the internal jugular vein(s) [1]. This septic thrombophlebitis often embolizes and affects multiple organ systems, most commonly the lungs [2-3]. Amongst the reported cases, 90% of patients are between the age of 19-22 with a worldwide incidence of 1/1,000,000 [1]. Lemierre’s syndrome is commonly caused by Fusobacterium necrophorum, though other bacteria have been indicated. Often referred to as a ‘forgotten disease’, Lemierre’s syndrome has seen a rise in cases over the years secondary to increased antibiotic resistance [2]. Clinicians should be aware of this very rare and deadly disease as prompt initiation of treatment is vital in patient outcomes. We describe an unusual case of a young female without any significant past medical history who presented with left-sided pleuritic chest pain several days after experiencing a sore throat. 

## Case presentation

A 21-year-old African American female without significant medical history presented to the emergency department with a one-day history of left-sided pleuritic chest pain and one episode of hemoptysis. She reported the pain was sudden in onset, 10/10 intensity, non-radiating, localized to the left lower chest wall, and aggravated by deep inspiration and movement but with no alleviating factors. The chest pain was associated with increasing shortness of breath. She had one episode of hemoptysis and described approximately two tablespoons of blood. She reported fever, sore throat, and left-sided neck pain four days prior to presentation, all of which resolved spontaneously. Her husband had a similar illness without any associated neck pain. She denied any recent travel, palpitations, nausea, vomiting, changes in bowel movements, or any IV drug use. Her family history was negative for any significant medical problems. She had never tested positive for a recent coronavirus disease 2019 (COVID-19) but was vaccinated x2 at that time. Vitals at presentation: blood pressure 94/58 mmHg, pulse 116 beats per minute, temperature of 97.7˚F, respiratory rate of 18, and oxygen saturation (SpO2) of 96%. 

**Figure 1:**
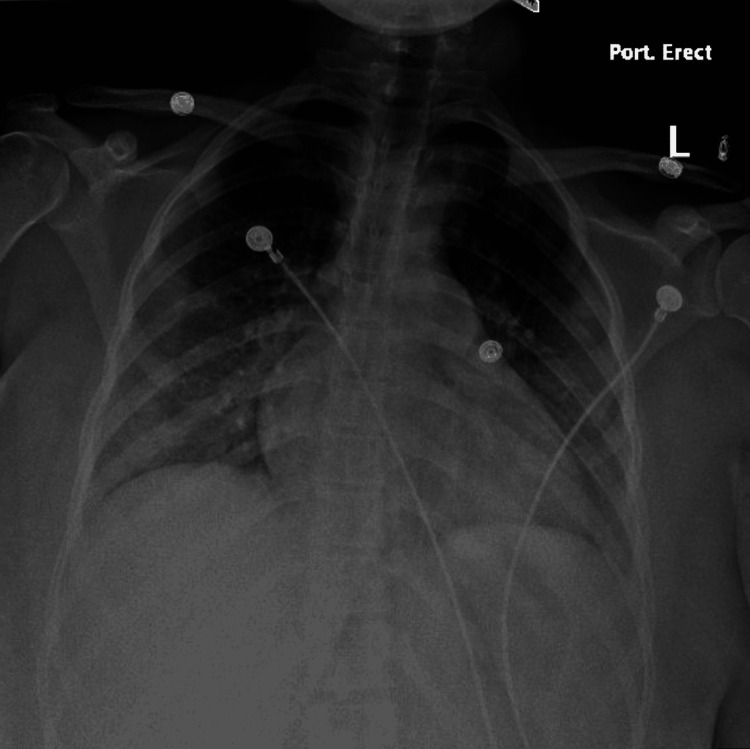
X-ray of the chest showing ill-defined ground-glass opacities in both lungs suspicious for pneumonia/pneumonitis.

**Figure 2:**
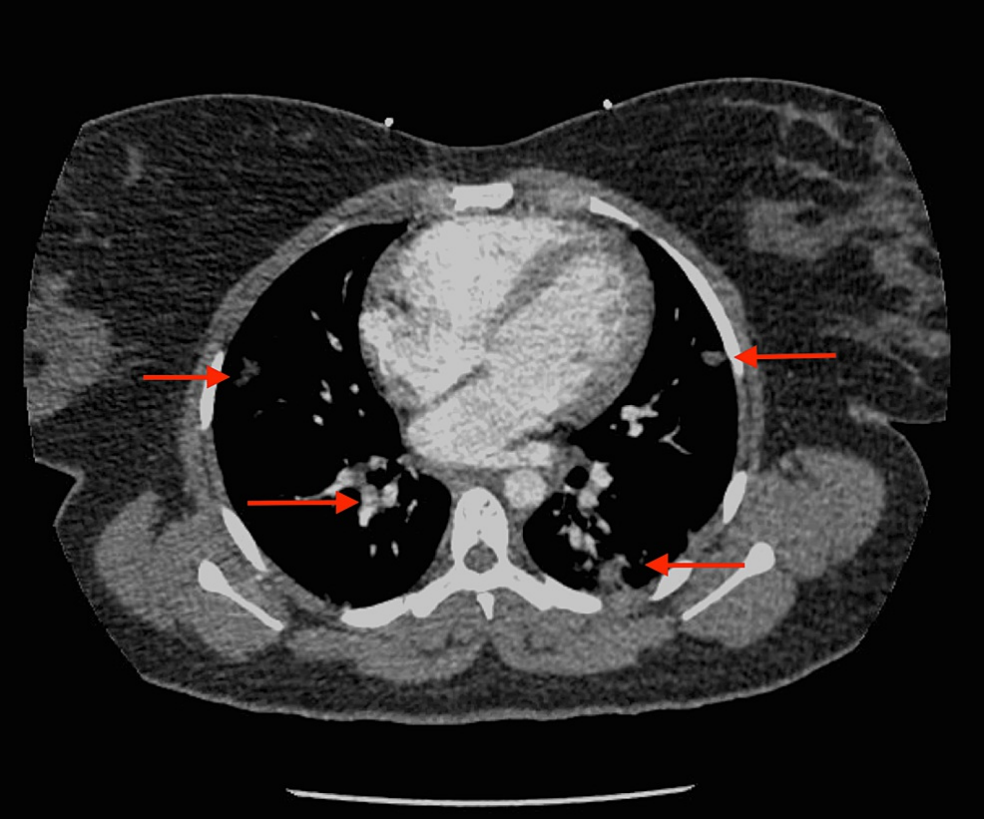
CT scan of the chest with arrows showing multiple bilateral pulmonary masses of varying sizes.

Upon physical examination, the patient was in acute distress, with tenderness on palpation to the left lower chest and found to be tachycardic and mildly tachypneic. Otherwise, at this time her physical examination was unremarkable. Laboratory findings on admission were significant for an elevated D-dimer, a low platelet count, an elevated alkaline phosphatase, elevated N-terminal pro-B-type natriuretic peptide (NT-proBNP), and elevated white blood cell count with increased bands, percent (Table 1). Labs at that time were otherwise within normal limits. Blood cultures were taken at that time which took several days to result. An X-ray of the chest showed ill-defined ground-glass opacities in both lungs suspicious for pneumonia/pneumonitis (Figure 1). A computed tomography (CT) scan of the chest was also negative for a large pulmonary embolism but showed multiple bilateral pulmonary masses of varying sizes, which were most prominent in the lower lung zones and concerning for an infectious etiology such as septic emboli (Figure 2).

**Table 1:** The patient's laboratory findings at presentation. NT-proBNP: N-terminal pro-B-type natriuretic peptide

Laboratory study	Results	References
D-Dimer (mg/L)	3.28 (mg/L)	< 0.50 (mg/L (FEU))
Platelet Count (10*3/uL)	66 (10*3/uL)	150 - 372 (10*3/uL)
Alkaline Phosphatase (U/L)	378 (U/L)	40 - 130 (U/L)
NT-proBNP (pg/mL)	1,239 (pg/mL)	< 300 (pg/mL)
White blood cell count (10*3/uL)	13.5 (10*3/uL)	3.6 - 11.0 (10*3/uL)
Bands, Percent (%)	15.0 (%)	0 - 6 (%)

Infectious disease consult was placed at that time and the patient was empirically started on piperacillin-tazobactam 3.375 g intravenously (IV) every eight hours, and vancomycin 1 g IV every 12 hours. The following day, she developed hemoptysis and increased shortness of breath. A CT abdomen and pelvis was performed and showed a small left pleural effusion with bibasilar lung consolidations and fatty infiltration of the liver. She now had an oxygen saturation of 75% on room air and required 6 liters of nasal cannula to increase her oxygen saturation to 94%. She was ultimately switched to a venturi mask at 6 liters of 35% fraction of inspired oxygen (FIO2). 

**Figure 3:**
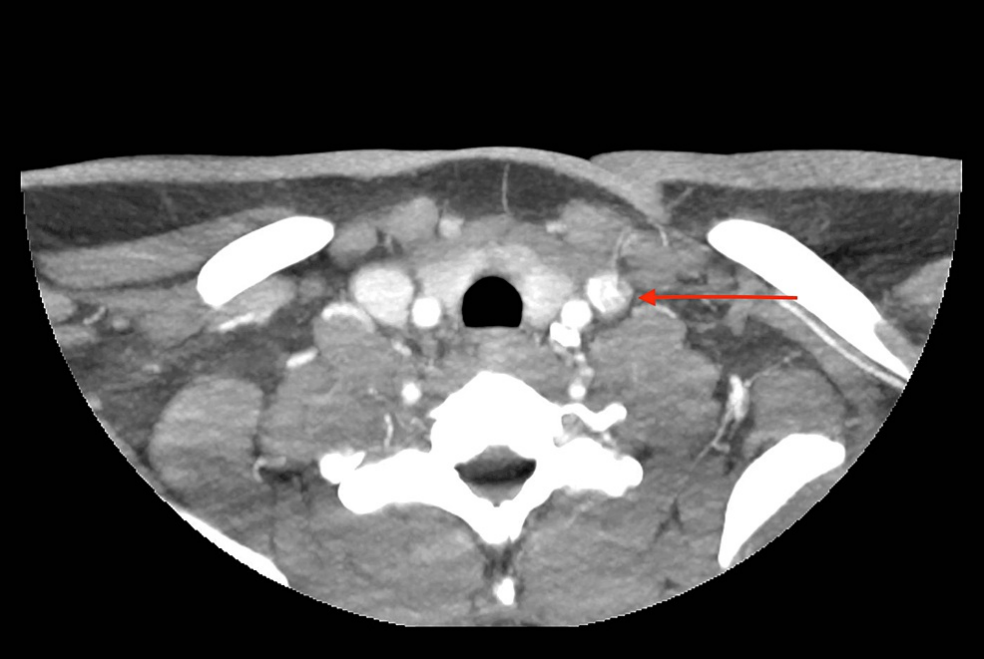
Arrow showing an ill-defined low-attenuation in the left internal jugular vein on CT angiogram of the head and neck.

At this time, blood cultures taken from admission had resulted and were positive for Fusobacterium necrophorum. The patient was worked up for Lemierre’s syndrome. A CT angiogram head and neck showed an ill-defined low-attenuation in the left internal jugular vein with a high clinical index of suspicion for intraluminal thrombus in this region (Figure 3). Further workup with an ultrasound (US) of the neck showed no evidence of internal jugular vein thrombus. The patient was started on treatment specific for Lemierre's syndrome; she was taken off of vancomycin, continued on piperacillin-tazobactam, and started on metronidazole as well as therapeutic heparin. Her hospital course was complicated and required several interventions. Due to worsening acute respiratory failure with hypoxia, the patient required intubation to protect her airways. She required bilateral thoracentesis, with pleural fluid showing no malignant cells, many neutrophils, but negative cultures. She later underwent bilateral video-assisted thoracic surgery with wash-out of left and right-sided empyema status post two left-sided chest tubes and one right-sided chest tube. She received a total of six weeks of antibiotic therapy with four weeks being intravenous. During the final two weeks of antibiotic treatment, she was discharged in stable condition on oral antibiotics. She also received a total of four weeks of therapeutic heparin before being discharged home.

## Discussion

Lemierre’s syndrome is a very rare and life-threatening complication of bacterial pharyngitis and tonsillitis. It is most commonly caused by Fusobacterium necrophorum, an obligate anaerobic, gram-negative bacilli, though other bacteria have been indicated [1]. It involves an extension of the infection with subsequent septic thrombophlebitis of the internal jugular vein(s) [1]. With a worldwide incidence of 1/1,000,000, Lemierre’s is incredibly rare but should not be forgotten, as prompt initiation of treatment is vital in patient outcomes [1]. Famously, in 1936, Lemierre himself reviewed 20 cases in the pre-antibiotic era. Of these 20 patients, 18 had fulminant and fatal evolution within 7-15 days of presentation [4]. With antibiotic resistance on the rise, due to widespread empiric coverage, clinicians should recognize the signs and presentation of Lemierre’s to improve patient outcomes with prompt diagnosis and appropriate intervention. 

Typically patients with Lemierre’s present with sore throat, fever, swollen or tender neck, evidence of metastatic lesions on imaging, and an identifiable source [5]. Our patient presented with pleuritic chest pain, tachycardia, one episode of hemoptysis, recent infection with associated neck swelling, and was found to have cardiomegaly on chest X-ray. Hemoptysis is a rare presentation of Lemierre's syndrome with one literature review only reporting hemoptysis in 8.2% of patients [5]. Several case reports have reported massive hemoptysis as a complication of Lemierre’s [6-9]. Though our patient produced only a small amount of blood when compared to other reports of hemoptysis, physicians should have high clinical suspicion when combined with the patient's history of presenting illness. Though only 8.2% of patients' courses are complicated by hemoptysis, 82.5% of patients experienced sore throats prior to presentation or concurrently [5]. Our patient experienced sore throat, self-resolving neck swelling, and pleuritic chest pain before she experienced hemoptysis. Her hospital course was marked by continued decompensation, requiring multiple surgical interventions. 

Another atypical presenting feature, in our patient, was cardiomegaly seen on chest X-ray. There have only been a few reported cases of cardiomegaly seen in Lemierre’s syndrome. Two of which were seen in two college students aged 19 and 21 [10-11]. Our patient did receive a workup for possible common etiologies of cardiomegaly. She reported no concerning family history of early cardiac failure, tested negative for HIV, denied any alcohol or recreational drug use, denied taking any medications, and showed no hilar lymphadenopathy or concerning signs or symptoms of sarcoid. The cardiomegaly was thought to be secondary to Lemierre’s syndrome and the underlying preceding infection. In a previously healthy patient, it was atypical to find cardiomegaly on X-ray. Our patient had no previous X-rays for us to compare to. 

The treatment for Lemierre’s is divided into three categories: antibiotics, anticoagulation, and other interventions such as surgery. Our patient required all three forms of treatment. She completed six weeks of antibiotics, and four weeks of anticoagulation and required multiple surgeries. The mean duration of antibiotic treatment in Lemierre’s is four weeks with a range of ten days to eight weeks [12]. Most cases of Lemierree’s caused by fusobacterium spp. respond well to carbapenem or piperacillin/tazobactam either as monotherapy or in combination with metronidazole [12]. Our patient was on combination therapy and responded very well. Our patient also responded well to four weeks of anticoagulation, though it is difficult to say whether or not this prevented further complications for her as she ultimately underwent several surgeries. Anticoagulation is still controversial with no definitive guidelines though one review found that 64% of patients with Lemierre’s had received anticoagulation [12]. This is not to say that patients receiving anticoagulation faired better. According to one meta-analysis, there was no statistically significant decrease in mortality with anticoagulation [13]. Due to septic emboli, surgical intervention is a common treatment in patients with Lemierre’s. In total, our patient underwent five surgeries which she tolerated very well. It is imperative that treatment is initiated promptly for better patient outcomes. With cases becoming more common, secondary to antibiotic resistance, clinicians should recognize the signs of Lemierre’s syndrome.

## Conclusions

Lemierre’s syndrome is an extremely rare complication of bacterial pharyngitis or tonsillitis that clinicians need to be aware of. With increasing antibiotic resistance due to widespread empiric coverage, there has been an increase in reported cases of Lemierre's syndrome. With the potential for multiple organ failure secondary to widespread septic emboli, Lemierre's syndrome can no longer be forgotten. Prompt initiation of treatment is needed for better patient outcomes. Antibiotics continue to be the mainstay treatment with surgical intervention being required for many patients. The need for anticoagulation continues to be debated for the treatment of Lemierre’s syndrome, though the majority of patients do receive some form of anticoagulation. As research continues, early studies suggest that anticoagulation is not statistically significant in reducing mortality. Our patient received all three modalities of treatment and responded very well and was discharged in stable condition after four weeks in the hospital.
